# A randomized clinical trial for the timing of tracheotomy in critically ill patients: factors precluding inclusion in a single center study

**DOI:** 10.1186/s13054-014-0585-y

**Published:** 2014-10-29

**Authors:** Antonio Diaz-Prieto, Antoni Mateu, Maite Gorriz, Berta Ortiga, Consol Truchero, Neus Sampietro, María Jesus Ferrer, Rafael Mañez

**Affiliations:** Servei de Medicina Intensiva, Hospital Universitari de Bellvitge, C/Feixa Llarga, Hospitalet de Llobregat, Barcelona, 08907 Spain; Departament de Gerencia, Hospital Universitari de Bellvitge, C/Feixa Llarga, Hospitalet de Llobregat, Barcelona, 08907 Spain

## Abstract

**Introduction:**

We investigated the potential benefits of early tracheotomy performed before day eight of mechanical ventilation (MV) compared with late tracheotomy (from day 14 if it still indicated) in reducing mortality, days of MV, days of sedation and ICU length of stay (LOS).

**Methods:**

Randomized controlled trial (RCT) including all-consecutive ICU admitted patients requiring seven or more days of MV. Between days five to seven of MV, before randomization, the attending physician (AP) was consulted about the expected duration of MV and acceptance of tracheotomy according to randomization. Only accepted patients received tracheotomy as result of randomization. An intention to treat analysis was performed including patients accepted for the AP and those rejected without exclusion criteria.

**Results:**

A total of 489 patients were included in the RCT. Of 245 patients randomized to the early group, the procedure was performed for 167 patients (68.2%) whereas in the 244 patients randomized to the late group was performed for 135 patients (55.3%) (*P* <0.004). Mortality at day 90 was similar in both groups (25.7% versus 29.9%), but duration of sedation was shorter in the early tracheotomy group median 11 days (range 2 to 92) days compared to 14 days (range 0 to 79) in the late group (*P* <0.02). The AP accepted the protocol of randomization in 205 cases (42%), 101 were included in early group and 104 in the late group. In these subgroup of patients (per-protocol analysis) no differences existed in mortality at day 90 between the two groups, but the early group had more ventilator-free days, less duration of sedation and less LOS, than the late group.

**Conclusions:**

This study shows that early tracheotomy reduces the days of sedation in patients undergoing MV, but was underpowered to prove any other benefit. In those patients selected by their attending physicians as potential candidates for a tracheotomy, an early procedure can lessen the days of MV, the days of sedation and LOS. However, the imprecision of physicians to select patients who will require prolonged MV challenges the potential benefits of early tracheotomy.

**Trial registration:**

Controlled-Trials.com ISRCTN22208087. Registered 27 March 2014.

**Electronic supplementary material:**

The online version of this article (doi:10.1186/s13054-014-0585-y) contains supplementary material, which is available to authorized users.

## Introduction

It is current medical practice in the ICU to perform a tracheotomy in patients undergoing prolonged mechanical ventilation (MV) [1]. However, there is no agreement on the optimal time for its performance. Several randomized clinical trials (RCT) [2–13] and four meta-analyses [14–17] have sought to demonstrate the benefits of early tracheotomy, and the results were either inconclusive or suggested a potential benefit for early tracheotomy in ICU survival but not in hospital survival. One of the main problems in the design of these studies is the difficulty for an earlier detection of patients who will require prolonged mechanical ventilation, due to the lack of obvious signs. As a result, the selection and inclusion of cases for these studies is based primarily on the clinical criteria of the attending physicians. This system for including patients in clinical trials may mask selection bias and mean that the samples used are unrepresentative of all patients admitted to the ICU requiring prolonged mechanical ventilation. In fact, two of the published studies mention the difficulty of selecting patients, due mainly to the doubts of the participating physicians regarding the patients’ need for tracheotomy [2,8].

The present study assessed the hypothesis that early tracheotomy primarily reduces 90-day mortality and secondarily the days of MV or sedation. We designed an RCT to evaluate the effect of early (before day 8) versus late (from day 14 onwards) tracheotomy on mortality in patients with 7 or more days of MV. All patients who met the inclusion criteria were consecutively included in an intention-to-treat analysis. However, prior to randomization, the attending physician was asked about the acceptance of the outcome of the randomization. Thus, we wanted to avoid selection bias. We anticipated that attending physicians would not accept the outcome of randomization of some patients and estimated 25% losses due to rejection of the randomization protocol. The study was stopped when we observed that losses or refusals were as high as 58%, and the results would not be representative.

According to these data, we also analyzed (*a posteriori*) the clinical differences between accepted and rejected patients in the RCT protocol, the cumulative incidence of tracheotomy in previously rejected patients and variables that determine the decision to perform tracheotomy in these patients.

## Materials and methods

### Study design

This was a prospective randomized trial conducted in four ICUs at Bellvitge University Hospital from 1 January 2006 to 28 February 2009. The study protocol was approved by the Ethics and Clinical Research Committee of Bellvitge University Hospital.

### Patient selection

All consecutive ICU-admitted patients were enrolled if they were mechanically ventilated for more than 48 h, were older than 18 years and written consent was available. To optimize the validity of the trial, patients were initially excluded only if they had undergone tracheotomy previously, were included in another trial or there was technical difficulty in performing percutaneous tracheotomy.

For the remaining patients we conducted a selection process for inclusion or exclusion, described in the next section. The first step was to select those patients who were expected to have more than 7 days ventilatory support. The second was to differentiate, according to the clinical judgment of the attending physician, patients having objective criteria contraindicating the performance of a tracheotomy according to the randomization protocol, from those in which the attending physician simply did not believe it would be required. The former were excluded and the procedure pursued for the selection of patients was as follows: 1) all patients with more than 48 h of MV were assessed; 2) between the third and fifth day of the MV, patient eligibility was assessed and written consent was obtained from relatives before inclusion of the patient in the study; 3) prior to randomization, we asked the attending physician if the duration of MV would likely be longer than 7 days. If the answer was affirmative, we asked if he/she would agree to apply the RCT protocol to the patient for performing tracheotomy. The reasons for rejection were categorized into one of four groups. Group 1 comprised critically ill patients without limitation of life support and any of the following conditions: intracranial hypertension (intracranial pressure (ICP) >15), risk of bleeding (platelets <50,000 or prothrombin time international normalized ratio (INR) >1.5), severe respiratory failure (positive end-expiratory pressure (PEEP) >10cmH_2_0 or PO_2_/FiO_2_ < 100). Group 2 comprised critically ill patient with poor prognosis with or without any type of decision to limit life support measures. Group 3 comprised patients in whom tracheotomy procedure could not be delayed on medical grounds (for example, extubation was not expected and unplanned extubation may have been life-threatening, or the neurological disease required an artificial airway long-term). Group 4 comprised patients in whom the medical decision to reject the result of the randomization was not due to any of the above reasons, and the physician preferred not to perform tracheotomy because extubation was expected within a few days and the physician therefore felt that it would not be necessary; 4) patients who were rejected by attending physicians for the RCT protocol classified into groups 1, 2 and 3 were excluded; and 5) patients who were accepted for implementation of the randomization protocol and rejected patients (classified into group 4) were included and randomized into a study design for intention-to-treat analysis.

We used a simple random sampling method. Randomization was performed using sealed opaque envelopes that hid the paper with the inscription of early or late. The envelopes were stored in a locked safe. Once the physician decided whether he would accept or reject the result of randomization, an envelope was chosen and opened for the result.

The tracheotomy was performed in accepted patients according to the result of randomization: early (before day 8) or late (from day 14 onwards of MV). In the remaining patients the decision on whether or not to perform a tracheotomy at a later date rested with the attending physician.

### Objectives

The primary end point was mortality at day 90. Secondary end points were the numbers of ventilator-free days between day 1 and day 28 and between day 1 and day 90, the length of stay in the ICU and the duration of sedation recorded as the number of days of intravenous sedatives in continuous perfusion.

### Tracheotomy procedure

All tracheotomies were performed using the percutaneous technique. In our hospital we have a team of three doctors with long experience of percutaneous tracheotomy, having performed more than 800 tracheotomy procedures before the start of the study [18]. Immediate procedure-related complications were reported.

### Mechanical ventilation features and weaning

Patients were ventilated with an initial tidal volume (Vt) of 6 to 8 mL/kg and not exceeding a plateau pressure of 30 cm H2O. The weaning was carried out by nursing staff in accordance with current recommendations [1], applying 1- to 2-hour-long disconnection tests with O2 administration via a T-piece on a daily basis. The decision to extubate was left to the attending physician. The extubation was only considered successful when the patient was discharged alive from the ICU.

### Sedation

Patients received continuous infusion sedation in accordance with the protocol in our hospital. We used two sedation regimens: one prolonged, with midazolam, morphine or fentanyl, and a shorter one with propofol and fentanyl or remifentanyl. The level of sedation was checked by nursing staff every 6 to 8 h in order to maintain a level between 3 to 4 on the Ramsay scale (awake and quiet or asleep but easily arousable). If sedation was insufficient (Ramsay 1) the doctor on duty was consulted to increase the dose. If the patient was sleeping heavily (Ramsay 5 to 6) doses were decreased gradually until stages 3 to 4 were reached. If medically prescribed, neuromuscular blockers were added.

### Data collection

Sociodemographic and clinical data were collected for all patients, including: simplified acute physiology score (SAPS), acute physiology and chronic health evaluation (APACHE), trauma injury severity score (TISS), sequential organ failure assessment (SOFA) on ICU admission and on the day of physician’s decision for participation in the RCT. We also collected medical decision accepting or rejecting randomization, the physician responsible for the decision, the day of the week that MV was started, the ICU length of stay and relevant therapeutic procedures that the patient was receiving, such as use of vasoactive drugs, parenteral nutrition, nitric oxide (NO), prone decubitus position, renal replacement techniques, circulatory assist devices, monitoring of ICP and cardiac output (Swan-Ganz catheter, Picco). Other data also recorded included MV-related data such as days of translaryngeal intubation (TLI), the application of the weaning protocol, episodes of extubation and reintubation, episodes of ventilator-associated pneumonia (VAP), type of sedation and days of sedation.

### Ventilator-associated pneumonia (VAP)

Clinical suspicion of pneumonia was recorded by the attending physicians on the basis of suggestive radiographic image, increased secretions, the presence of fever, leukocytosis or leucopenia and hypoxemia [19,20]. The diagnosis of VAP had to be confirmed by positive cultures in bronchoscopic bronchoalveolar lavage (BAL) or blind BAL. The diagnostic thresholds of BAL and blind BAL for the consideration of VAP were 10^4^ and 10^5^ cfu/mL respectively.

### Statistical analysis

The RCT was designed on the basis of previous data from 1,000 patients receiving mechanical ventilation for more than 7 days, with a 90-day mortality of 41%. We calculated that to reduce the mortality from 41% to 30% and to achieve a power of 80% with a Type I error of 0.05, 544 patients were required in each group, or 726 calculating a loss (rejection rate) of 25% of patients.

Categorical variables were described in the univariate and bivariate analysis using the overall number of cases (n) and the percentage of each category. For continuous variables we used the median (range) due to lack of normality. For bivariate analysis, we used the analysis of variance (ANOVA) test to compare means and the non-parametric Kruskal-Wallis test. All comparisons were unpaired and all tests of significance were two-tailed.

The primary analysis consisted of evaluating the effect of tracheotomy on the primary outcome (that is, 90-day mortality), with adjustment by means of a Cox multivariate proportional-hazards model. We used logistic regression analysis to estimate the adjusted odds ratio of those variables associated with acceptance or rejection in the RCT due to the physician’s decision and for receiving a tracheotomy in patients rejected from the RCT. A stepwise approach was used to enter new terms into the logistic regression model. The limit for the acceptance or removal of the new terms was set as 0.05. Variables with *P* <0.15 were entered into the multivariate analysis. Results of the logistic regression analysis are reported as adjusted odds ratios and their 95% confidence intervals. The significance level was set at *P* <0.05 throughout. All statistical analyses were conducted using the Statistical Software Program (SPSS, Chicago, IL, USA).

## Results

**Figure 1:**
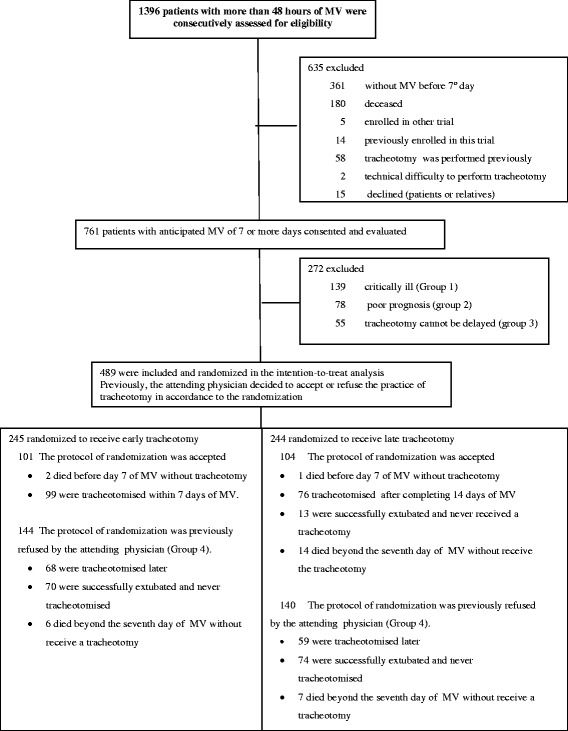
**Patient flow diagram.** MV, mechanical ventilation.

During the study period 3,152 consecutive patients underwent MV. Of these, 1,396 (44%) with more than 48 h on MV were evaluated for inclusion in the RCT (see the flow diagram in Figure 1). In total, 489 patients were included in the analysis: 245 in the early group and 244 in the late group. Before knowing the result of the randomization, 284 (58%) patients were refused by the attending physician to undergo a tracheotomy and 205 (42%) patients were accepted. The latter integrated the per-protocol analysis.

Of 245 patients randomized to the early tracheotomy group, the procedure was performed in 167 patients (68.2%; 95% CI, 62%, 74%) whereas in the patients randomized to the late group was performed for 135 patients (55.3%; 95% CI, 49%, 62%) (*P* <0.004). Eight cases randomized to early group (3.2%; 95% CI, 1.4% to 6.3%) died without tracheotomy, whereas 22 cases in the late group died without tracheotomy (9%; 95% CI, 6%, 13%) (*P* <0.009). Finally, 70 patients randomized to the early group were successfully extubated and did not require tracheotomy (28.6%; 95% CI, 23%, 35%), whereas 87 patients in the late group were successfully extubated (35.7%; 95% CI, 30%, 42%) (*P* <0.094).

Characteristics of the patients at inclusion in the study were similar in the two groups except for the number of cases with intracranial pressure monitoring that was higher in the early group. Related differences were observed in trauma patients or comatose patients as a cause of MV. The rate of rejected cases was similar in the two groups (58.8% and 57.4% in the early and late groups, respectively), as occurred with the severity scores and SOFA measurements (Table 1).

**Table 1:** **Baseline and clinical characteristics of patients at inclusion in the study**

	**Early n = 245**	**Late n = 244**	***P*** **-value**
Rejected by attending physician	144 (59%)	140 (57%)	0.7540
Sex, male	170 (69%; 63 to 75)	159 (65%; 59 to 71)	0.3196
Age	64 (18 to 89)	65.5 (19 to 88)	0.7814
SAPS 2	38 (3 to 78)	37.5 (10 to 85)	0.6272
Probability of death (SAPS 2)	0.21 (0.00 to 0.91)	0.21 (0.01 to 0.95)	0.6315
SAPS 3	62 (31 to 114)	61 (29 to 105)	0.8088
Probability of death (SAPS 3)	0.40 (0.02 to 0.96)	0.38 (0.02 to 0.93)	0.8108
APACHE II	20 (5 to 40)	19 (4 to 38)	0.2353
ISS, n	29 (9 to 66) [32]	30 (25 to 59) [20]	0.6165
SOFA admission	9 (1 to 19)	9 (1 to 20)	0.8665
SOFA decision	6 (1 to 17)	6 (0 to 15)	0.6943
Difference between SOFA	2 (−7 ; 14)	3 (−5 ; 11)	0.6100
Elective surgery	69 (28%; 22 to 34)	61(25%; 20 to 31)	0.4285
Trauma	32 (13%; 9 to 18)	20 (8%; 5 to 12)	0.0810
Emergency surgery	43 (18%; 13 to 24)	57 (23%; 18 to 29)	0.1113
Medical condition	101 (41%; 35 to 48)	106 (44%; 38 to 51)	0.6196
Inhaled nitric oxide	20 (8%; 6 to 20)	21 (9%; 6 to 21)	0.8596
Prone decubitus	17 (7%; 4 to 17)	25 (10%; 7 to 21)	0.1919
Swan-Ganz catheter	37 (15%; 10 to 26)	32 (13%; 10 to 25)	0.5159
Renal replacement techniques	35 (14%; 4 to 17)	37 (15%; 11 to 26)	0.7841
Vasoactive drugs	204 (83%; 74 to 90)	213 (87%; 84 to 96)	0.2086
Parenteral nutrition	70 (29%; 17 to 35)	76 (31%; 16 to 34)	0.5337
Intracranial pressure monitoring	35 (14%; 10 to 26)	19 (8%; 3 to 15)	0.0219
Circulatory assist	15 (6%; 2 to 12)	15 (6%; 2 to 12)	0.9908
**Main reason for ventilatory support**			
Acute respiratory insufficiency	167 (68%; 62 to 74)	176 (72%; 66 to 78)	0.3703
Neuromuscular illness	5 (2%; 0.7 to 5)	4 (2%; 0.7 to 5)	0.7365
Coma (Glasgow coma score <10)	60 (24%; 19 to 30)	46 (19%, 14 to 24)	0.1241
Decompensated COPD	11 (5%; 3 to 8)	14 (6%; 3 to 10)	0.5379
Acute asthma attack	1	1	
Other respiratory disease	1	3	

Results of the quantitative variables are expressed in medians and ranges. Qualitative variables are expressed in percentages and their 95% CI. COPD, chronic obstructive pulmonary disease; ISS, injury severity score (only evaluated on trauma patients); SAPS, simplified acute physiology score; SOFA, sequential organ failure assessment; APACHE, acute physiology and chronic health evaluation.

### Primary and secondary outcomes

Mortality at day 90 was similar in the early and the late group: 25.7% (63 of 245 participants) versus 29.9% (73 of 244) (*P* = 0.2996). After adjustment for the intracranial pressure monitoring and diagnostic category, mortality remained similar in both groups. The duration of sedation was shorter in the early group with a median (range) of 11 (2 to 92) days, compared to 14 (0 to 79) days in the late group (*P* <0.02). There were nearly significant between-group differences in the numbers of ventilator-free days (Table 2). The length of stay in ICU did not differ significantly between the two groups. Similarly, the duration of intubation in patients successfully extubated, the rate of application of the weaning protocol, the rate of reintubation and the VAP rate, did not differ significantly between the two groups.

**Table 2:** **Primary and secondary outcomes**

	**Early N = 245**	**Late N = 244**	***P*** **-value**
28-day mortality	42 (17%; 13 to 22)	47 (19%; 14 to 25)	0.5436
90-day mortality	63 (26%; 20 to 31)	73 (30%;24 to 36)	0.2996
Hospital mortality	67 (27%; 22 to 33)	78 (32%; 26 to 38)	0.2634
Days free of mechanical ventilation, 28-day	11 (0, 22)	9 (0, 22)	0.0529
Days free of mechanical ventilation, 90-day	73 (0 to 84)	71 (0 to 84)	0.0546
Duration of sedation, days, n	11 (2 to 92)	14 (0 to 79)	0.0194
ICU length of stay, days, n	22 (6 to 101)	22.5 (6 to 174)	0.3088
**Other characteristics related to mechanical ventilation and tracheotomy**			
Weaning protocol applied in intubated patients	125 (51%; 45 to 57)	141 (58%; 52 to 64)	0.1331
Failed extubation (reintubated)	38 (16%; 11 to 21)	27 (11%; 7 to 16)	0.1477
Days of intubation in tracheotomized patients	7 (6 to 29)	14 (9 to 47)	0.0009
Days of intubation in successfully extubated patients	10 (8 to 18)	10 (8 to 21)	0.8921
Patients sedated, n (%)	234 (96%; 92 to 98)	235 (96%; 93 to 98)	0.6546
Deep sedation with neuro-blocker drugs	36 (15%; 11 to 20)	52 (21%; 16 to 27)	0.0568
Maximum positive end-expiratory pressure	7 (0 to 16)	7 (3 to 16)	0.9473
Ventilation-associated pneumonia	33 (13%; 9 to 19)	23 (9%; 6 to 15)	0.1604
Early complications of tracheotomy	2 (1.2%: 0.2 to 4)	9 (6.7%; 3 to 12)	0.0139

Results of the quantitative variables are expressed in medians and ranges. Qualitative variables are expressed in percentages and their 95% CI.

For the 302 patients receiving tracheotomy, procedure-related complications were reported for a total of 11 patients (3.6%; 95% CI 1.8%, 6.4%): 2 of 167 patients (1.2%) in the early group (95% CI 0.2%, 4.3%) and 9 of 135 patients (6.7%) in the late group (95% CI, 3.1%, 12.3%) (*P* <0.05). The most frequent complication was bleeding sufficient to require hemostatic measures or another intervention, which occurred in 10 cases (3.3%), though no case required surgical intervention. One patient suffered a pneumomediastinum.

### Per protocol analysis

A total of 205 cases were accepted by attending physicians to undergo a tracheotomy according to the result of randomization. Of these, 101 patients were allocated to early and 105 were allocated to the late tracheotomy group. In these patients, the procedure was conducted as per protocol, either on day 7 or after day 14, or never, due to no longer being clinically indicated.

The tracheotomy was performed in 99 patients in the early group (98%; 95% CI 93%, 99.8%) whereas it was performed in 76 patients in the patients randomized to the late group (73.1%; 95% CI 63%, 81%) (*P* <0.001). Two cases randomized to early group (2%; 95% CI 0.2%, 7%) dead without tracheotomy, whereas 15 cases in the late group died without tracheotomy (14.4%; 95% CI 8%, 23%) (*P* <0.002). Nobody included in the early group, was extubated before day 7. Thirteen patients randomized to the late group were successfully extubated and never required tracheotomy (12.5%; 95% CI, 7%, 20%).

Baseline characteristics of these patients can see in Table 3. Characteristics of the patients at inclusion in the study were similar in the two groups except for the number of cases with ICP monitoring, which was higher in the early group. The crude 90-day mortality was 29.7% (95% CI, 21%, 40%) in the early group and 42.3% (95% CI, 33%, 52%) in the late group (*P* = 0.0603). The Cox regression model yielded a hazard ratio for death at 90 days in the early group, as compared with the late group, of 0.94 (95% CI, 0.59, 1,51; *P* = 0.4706), after adjustment for the intracranial pressure monitoring. The early group showed more ventilator-free days at 28 and 90 days, lesser duration of sedation and the ICU length of stay than the late group (Table 4).

**Table 3:** **Per-protocol analysis of randomized patients accepted by attending physicians**

	**Early n = 101**	**Late n = 104**	***P*** **-value**
Sex, male	73 (72%; 63 to 81)	63 (61%; 50 to 70)	0.0763
Age, years	65 (19 to 84)	68.5 (20 to 88)	0,3229
SAPS 2	38 (9 to 78)	37 (10 to 85)	0.8156
Probability of death (SAPS 2)	0.21 (0.01, 0.91)	0.20 (0.01, 0.95)	0.8083
SAPS 3	58 (31 to 114)	60 (32 to 105)	0.6906
Probability of death (SAPS 3)	0.32 (0.02, 0.96)	0.36 (0.02, 0.93)	0.6932
APACHE II	20 (7 to 37)	19 (8 to 38)	0.5508
ISS, n	29 (16 to 57) [17]	31 (25 to 59) [10]	0.8204
SOFA admission	8 (1 to 17)	9 (1 to 20)	0.3666
SOFA decision	6 (2 to 17)	7 (2 to 15)	0.5511
Difference between SOFA	2 (−7 ; 9)	2 (−5 ; 10)	0.2849
Elective surgery	34 (33.7%; 25 to 44)	32 (30.8%; 22 to 41)	0.6575
Trauma	17 (16.8%; 10 to 26)	10 (9.6%; 5 to 17)	0.1266
Emergency surgery	12 (11.9%; 6 to 20)	21 (20.2%; 13 to 29)	0.1055
Medical condition	38 (37.6%; 28 to 48)	41 (39.4%; 30 to 50)	0.7913
Inhaled nitric oxide	12 (12%; 6 to 20)	13 (12.5%; 6 to 19)	0.8923
Prone decubitus	9 (9%; 4 to 16)	13 (12.5%; 7 to 21)	0.4065
Swan-Ganz catheter	17 (17%; 10 to 26)	17 (16%; 10 to 25)	0.9003
Renal replacement techniques	10 (10%; 4 to 17)	18 (17%; 11 to 26)	0.1226
Vasoactive drugs	84 (83%; 74 to 90)	95 (91%; 84 to 96)	0.0786
Parenteral nutrition	26 (26%; 17 to 35)	25 (24%; 16 to 33)	0.7778
Intracranial pressure monitoring	17 (17%; 10 to 26)	8 (8%; 3 to 15)	0.0456
Circulatory assist	6 (6%; 2 to 12)	6 (6%; 2 to 12)	0.9583
**Main reason for ventilatory support**			
Acute respiratory insufficiency	68 (67.3%; 56 to 76)	72 (69.2%; 59 to 78)	0.7696
Neuromuscular illness	3 (3%; 0.6, 9)	2 (1.9%; 0.2, 7)	0.6270
Coma (Glasgow coma scale <10)	27 (26.7%; 19 to 37)	20 (19.2%; 12 to 28)	0.2014
Decompensated COPD	3 (3%; 0.6, 9)	9 (8.6%; 4 to 16)	0.0831
Acute asthma attack	0	0	
Other respiratory disease	0	1	

Baseline and clinical characteristics at inclusion in the study. Results of the quantitative variables are expressed in medians and ranges. Qualitative variables are expressed in percentages and their 95% CI. COPD, chronic obstructive pulmonary disease; ISS, injury severity score (only evaluated on trauma patients); SAPS, simplified acute physiology score; SOFA, sequential organ failure assessment; APACHE, acute physiology and chronic health evaluation.

**Table 4:** **Per-protocol analysis: primary and secondary outcomes**

	**Early n = 101**	**Late n = 104**	***P*** **-value**
28-day mortality	20 (20%; 13 to 29)	30 (29%; 20 to 38)	0.1317
90-day mortality	30 (30%; 21 to 40)	44 (42%; 33 to 52)	0.0603
Hospital mortality	31 (31%; 22 to 41)	45 (43%; 34 to 53)	0.0623
Days free of mechanical ventilation, 28-day	8 (0, 22)	4 (0, 22)	0.0003
Days free of mechanical ventilation, 90-day	70 (24 to 84)	66 (0 to 84)	0.0002
Duration of sedation	11 (2 to 66)	16.5 (5 to 63)	0.0006
ICU length of stay	24 (6 to 84)	29 (6 to 174)	0.0276
**Other characteristics related to mechanical ventilation and tracheotomy**			
Weaning protocol applied in intubated patients	16 (16%; 9 to 24)	27 (26%; 18 to 36)	0.0752
Failed extubation (reintubated)	13 (13%; 6 to 20)	11 (11%; 5 to 18)	0.6095
Days of intubation in tracheotomized patients	7 (5 to 7)	14 (14 to 22)	0.0000
Days of intubation in successfully extubated patients	0	14 (11 to 17)	
Patients sedated, n, %	95 (94%; 87 to 98)	102 (98%; 93 to 99)	0.1375
Deep sedation with neuroblocker drugs	19 (19%; 12 to 28)	27 (26%; 18 to 36)	0.2199
Maximum positive end-expiratory pressure	7 (2 to 16)	7 (3 to 15)	0.8966
Ventilator-associated pneumonia	11 (11%; 6 to 19)	12 (12%; 6 to 19)	0.8833
Early complications of tracheotomy	2 (2%;0.3-7)	5 (6.6%; 1.5-11)	0.2417

Results of the quantitative variables are expressed in medians and ranges. Qualitative variables are expressed in percentages and their 95% CI.

### Comparison between patients accepted and patients rejected for the RCT protocol (group 4)

We compared clinical characteristics from patients accepted for RCT and those rejected for medical decision (Additional file 1). Patients accepted for RCT were older, had higher 90-day mortality and ICU stay, though severity on admission (SAPS and APACHE scores) was similar. The groups differed in terms of evolution: clinical improvement (difference in SOFA score) was significantly higher in patients rejected for RCT. The two groups also showed differences regarding elective surgery as diagnostic category and use of nitric oxide that were higher in patients accepted. The number of tracheotomies, days of intubation in extubated patients, days on MV, days of sedation and use of neuromuscular blocking agents were higher in patients accepted for RCT, whilst the number on weaning protocols, patients extubated and days free of MV were higher in patients rejected for RCT (Additional file 1: Table S1 and S2). In logistic regression analysis (Table 5) using the sample of these two groups and analyzing the acceptance or rejection for the RCT as the dependent variable, we found that older age, a negative difference in the SOFA score, the use of neuromuscular blockers and the use of nitric oxide were the independent variables that increased the likelihood of being accepted on the RCT. Admission on the weekend increased the likelihood of rejection.

**Table 5:** **Variables independently associated with the physician decision to accept or reject the participation in the RCT protocol: logistic regression analysis**

**Variable**	**Adjusted odds ratio**	**95% CI**	***P*** **-value**
Sequential organ failure assessment, difference	0.85	0.79 to 0.91	0.001
Weekend admission	0.47	0.26 to 0.85	0.012
Neuromuscular blocker use	1.90	1.11 to 3.25	0.018
Age	1.01	1.00 to 1.03	0.010
Inhaled nitric oxide	1.98	0.95 to 4.09	0.066

### Analysis of factors associated with tracheotomy in patients rejected (group 4)

Of the 284 patients initially rejecting the RCT protocol, 127 (45%) underwent tracheotomy at a median of 14 days from intubation (range 9 to 47 days). We also evaluated factors associated with the performance of tracheotomy in patients initially rejected (Table 6). The independent variables that increased the likelihood for a patient eventually undergoing a tracheotomy after being initially rejected were: failure of extubation, the presence of VAP, age, and worsening of the SOFA score after the start of the MV.

**Table 6:** **Variables independently associated with tracheotomy in the patients rejected for the RCT: logistic regression analysis**

**Variable**	**Adjusted odds ratio**	**95% CI**	***P*** **-value**
Reintubation	22.96	6.73 to 78.27	0.001
Sequential organ failure assessment, difference	0.88	0.79 to 0.97	0.012
Ventilator-associated pneumonia	2.30	0.98 to 5.39	0.055
Age	1.02	1.00 to 1.03	0.058

## Discussion

The results of this study show that early tracheotomy reduces the days of sedation. It also suggests that it has no influence on mortality, but the clinical trial is underpowered preventing conclusions to this regard or other. For those patients accepted by attending physicians to receive the tracheotomy according to the result of randomization, the days of ventilatory support, sedation and the length of stay in ICU were significantly lower in the early tracheotomy group than in the late group. In addition, more tracheotomies were performed in the early group than in the late group. This is due in part to the fact that more patients died in the late group without receiving the procedure, or were extubated without tracheotomy. The results confirm those from other studies [11,12], and highlights the risk of performing unnecessary tracheotomies if this procedure is performed within one week of mechanical ventilation, as two recent multicenter RCTs have also shown [9,13].

There are no objective criteria to foresee patients who will require prolonged ventilatory support and therefore a tracheotomy. Physicians base their decision to perform tracheotomy, on subjective clinical criteria that are formed along the patient’s progress, in some cases before and in others later. This raises two issues in this kind of study. One is the possibility of performing unnecessary tracheotomies. The other is not to include patients who are subsequently tracheotomized. Our study was designed to include consecutive patients with 7 days of MV (as potential candidates for a tracheotomy) in an intention-to-treat analysis, but respecting the decision of the attending physician about the future need for a tracheotomy in each patient. To avoid bias in the selection (changes in the decision after knowing the result of the randomization), we invited the physician to consider if the patient could need a tracheotomy before knowing the outcome of the randomization. In all cases with a positive answer, the tracheotomy was performed according to randomization. The problem was that in 58% of patients, much more than estimated, physician refused the tracheotomy according to randomization, and that 45% of these underwent the procedure later.

The study design we used to select and include cases is indeed arguable, since not all consecutive patients with 7 days of ventilatory support will require a tracheotomy. However, the medical judgment about the likely duration of MV does not guarantee proper patient selection either [21]. In our study we performed tracheotomies in 68% of patients randomized to the early group (40% for accepted and 28% for rejected patients) and 55% in the late group (31% for accepted and 24% for rejected patients). Therefore, the percentage of tracheotomies in the early group was reduced compared to other studies in which the percentage reached 84.6% of patients, though in the latter case the procedure was performed within 4 days of ICU admission [13]. The sooner taken the decision to select a patient to perform tracheotomy, the more difficult it is to foresee the duration of the MV, and the probability of performing unnecessary tracheotomies will also be higher.

Several authors have complained about the difficulty in selecting patients for the time of tracheotomy in mechanically ventilated patients [2,8], although no studies have shown what happens to those patients who are eligible but are not included. Blot *et al*. [8] sent a questionnaire to all the investigators at the end of the study and it revealed that only 10% to 20% of the patients assessed for eligibility were actually included. So, it becomes very difficult to compare results from different studies because depending on the way of selection, the patient cohort may differ in its characteristics. Thus, Blot *et al*. included patients in the RCT based on expected duration of MV >7 days and the clinical judgment of the investigators. The results showed lower mortality in patients on MV for more than 7 days than in those used to calculate the sample [8]. This was attributed to selection bias in favor of the inclusion of patients with a better prognosis. In our study the mortality rate of the sample used for the calculation of the population was the same that in Blot’s study. However, the mortality of our accepted patients was 35%, higher than the 20% that they reported, indicating that physician selection criteria were different between the two studies.

Due to the high number of rejections of the randomization protocol, we analyzed the differences between accepted and rejected patients. Most of the factors that increased the likelihood of being accepted in the RCT indicated a clinical worsening from admission. These included impairment of the SOFA score, neuromuscular blockers administration, older age, use of nitric oxide and admission on a weekday. The latter probably reflects a shorter time of care by the attending physician from admission to the time of decision to inclusion in the RCT when patients were admitted during the weekend compared to weekdays. Selection of patients with worsened clinical condition after ICU admission for the RCT is similar to that used by Terragni *et al*. [9] in another study about the timing of tracheotomy in MV. They considered a SOFA score of 5 and a worsening of the PaO2/FiO2 to select patients according to their severity. Interestingly, in the latter study early tracheotomy was associated with fewer days of MV and ICU admission. These results are similar to those found in our RCT that included patients who did not improve in the initial days after ICU admission by medical selection. Despite the potential benefits, Terragni *et al*. concluded that earlier tracheotomy did not provide advantages. The reason is that the study did not result in statistically significant reduction in incidence of VAP, which was the main goal of the study, and increased the number of patients who received tracheotomy by 12%.

By not accepting participation in our RCT attending physicians prevented 28% of tracheotomies in those patients that would be assigned to the early tracheotomy group, but delayed the other 28%. The odds of undergoing a tracheotomy in these circumstances increased with the need for reintubation, the presence of VAP, age, and clinical worsening as indicated by the SOFA score. Reintubation and VAP are complications associated with prolonged MV and have been related to the practice of tracheotomy in previous studies [22,23]. However, the particular issue of VAP should be taken carefully because the diagnosis may be subjected to bias in unblinded RCTs [24].

This study has several limitations. The most important is the discontinuation before reaching the expected number of cases due to the high number of rejections to the randomization protocol that caused a notable selection bias, leading to underpowered results. In addition, the external validity of this study is limited by being a single-center study. However, it is a large consecutive sample of critically ill patients with 7 days of ventilatory support, including broad medical and surgical conditions. The study was not originally designed with the intention to explore the potential barriers to include patients for tracheotomy, though we established classification groups in the study design to identify the main causes and the number of rejected patients. Subsequent analysis of the characteristics and differences between accepted and rejected patients is therefore limited. We found some characteristics that define the accepted patients such as an unfavorable clinical course, with respiratory or other type of complications that impact on the SOFA score during their evolution. However, there are probably others that we were not able to detect, both clinical (related to the disease or the clinical course), as related to the organization or the beliefs and perceptions of attending physicians. Further research should try to establish a better definition of these patients while minimizing the impact of subjective physician’s opinion. If this is achieved, the benefits of performing an early tracheotomy may counteract the risks of practicing unnecessary procedures and associated complications.

## Conclusions

This trial shows that early tracheotomy reduced the days of sedation in consecutive critically ill patients, requiring 7 or more days of MV, but was underpowered to provide new information about the influence on mortality in these patients. In those patients selected by their attending physicians during the first week of MV as potential candidates for a tracheotomy, an early procedure lessened the days of MV, the days of sedation and ICU length of stay. However, the imprecision of physicians to select patients who will require prolonged MV challenges the potential benefits of early tracheotomy due to the risk of performing unnecessary procedures.

## Key messages

- Early tracheotomy reduces the days of sedation of patients with seven or more days of ventilatory support
- Early tracheotomy may lead to unnecessary procedures due to the limited medical capacity to predict within the first week, those patients that may require prolonged ventilatory support
- Critically ill patients selected by attending physician during the first week of MV as candidates to receive a tracheotomy are characterized by an unfavorable clinical course and may benefit from early tracheotomy

## Additional file

Additional file 1:
**Comparison between patients accepted or rejected (group 4), clinical characteristics and results.**

## Abbreviations

AP: attending physician
APACHE: acute physiology and chronic health evaluation
BAL: bronchoscopic bronchoalveolar lavage
CFU: colony-forming unit
COPD: chronic obstructive pulmonary disease
GCS: Glasgow coma score
HUB: *Hospital Universitari Bellvitge*
ICP: intracranial pressure
INR: international normalized ratio
ISS: injury severity score
LOS: length of stay
MV: mechanical ventilation
NO: nitric oxide
PEEP: positive end-expiratory pressure
RCT: randomized clinical trial
SAPS: simplified acute physiology score
SOFA: sequential organ failure assessment
TLI: translaryngeal intubation
VAP: ventilator-associated pneumonia
Vt: tidal volume
