# Supplementary material for: A randomized clinical trial for the timing of tracheotomy in critically ill patients: factors precluding inclusion in a single center study
Source: Crit Care. 2014 Oct 29;18(5):585. doi: 10.1186/s13054-014-0585-y (PMC4234827; doi:10.1186/s13054-014-0585-y)
Supplement: Additional file 1: — Comparison between patients accepted or rejected (group 4), clinical characteristics and results. [file 13054_2014_585_MOESM1_ESM.doc]

**Additional file 1**

**Table S1.** **Comparison of clinical characteristics of patients accepted and rejected (group 4).** Results of the quantitative variables are expressed in medians and ranges. Qualitative variables are expressed in percentages and their 95% confidence intervals. ISS: Injury Severity Score (only evaluated on trauma patients). SAPS: Simplified Acute Physiology Score. SOFA: Sequential Organ Failure Assessment. APACHE: Acute Physiology and Chronic Health Evaluation .

|  | **ACCEPTED** | **REJECTED** | **p value** |
| --- | --- | --- | --- |
| Nº | 205 (42%) | 284 (58%) |  |
| 28-day mortality | 50 (24.4%; 19-31) | 39 (13.7%; 10-18 ) | **0.0026** |
| 90-day mortality | 74 (36.1%; 29-43) | 62 (21.8%; 17-27 ) | **0.0005** |
| Hospital mortality | 76 (37%; 30-44) | 69 (24.3%; 19-30 ) | **0.0023** |
| Sex (male) | 136 (66.3%; 59-73) | 193 (68%; 62-73) | 0.7070 |
| Age | 67 (19, 88) | 62 (18, 89) | **0.0162** |
| ICU length of stay | 26 (6, 174) | 19 (6, 135) | **0.0007** |
| SAPS 2 | 37 (9, 85) | 38 (3, 75) | 0.8758 |
| Probability of death (SAPS 2) | 0.20 (0.01, 0.95) | 0.21 (0.00, 0.89) | 0.8806 |
| SAPS 3 | 60 (31, 114) | 63 (29, 99) | 0.1352 |
| Probability of death (SAPS 3) | 0.36 (0.02, 0.96) | 0.42 (0.02, 0.91) | 0.1351 |
| APACHE II | 19 (7, 38) | 20 (4, 40) | 0.4185 |
| ISS [nº] | 30 (16, 59) [27] | 30 (9, 66) [25] | 0.7826 |
| SOFA on admission | 8 (1, 20) | 9 (2, 19) | 0.2507 |
| SOFA at the time of medical decision | 6 (2, 17) | 5 (0, 15) | **0.0007** |
| Difference between SOFA | 2 (-7 ; 10) | 3 (-5 ; 14) | 0.0007 |
|  |  |  |  |
| Elective surgery | 66 (32.2%; 26-39) | 64 (22.5%; 18-28) | **0.0170** |
| Trauma | 27 (13.2%; 9-19) | 25 (9%; 6-13) | 0.1221 |
| Emergency surgery | 33 (16.1%; 11-22) | 67 (23.5%; 18-28) | **0.0426** |
| Medical condition | 79 (38.5%; 31-45) | 128 (45%; 39-51) | 0.1490 |
| Inhaled nitric oxide | 25 (12%; 8-17) | 16 (6%; 3-9) | **0.0098** |
| Prone decubitus | 22 (11%; 7-16) | 20 (7%; 4-11)) | 0.1508 |
| Swan-Ganz catheter | 34 (17%; 12-23) | 35 (12%; 8-17) | 0.1744 |
| Renal replacement techniques | 28 (14%; 9-19) | 44 (15.5%; 11-20) | 0.5722 |
| Vasoactive drugs | 179 (87%; 81-91) | 238 (84%; 79-88) | 0.2792 |
| Parenteral nutrition | 51 (25%; 19-31) | 95 (33%; 28-39) | **0.0410** |
| Intracranial pressure monitoring | 25 (12 %; 8-18) | 29 (10%; 7-14) | 0.4898 |
| Circulatory assist | 12 (6%; 3-10) | 18 (6%; 4-10) | 0.8257 |

**Table S2. Comparison of characteristics related to MV and the tracheotomy of randomized patients that were accepted or rejected (group 4).** Results of the quantitative variables are expressed in medians and ranges. Qualitative variables are expressed in percentages and their 95% confidence intervals.

|  | **ACCEPTED**  **N=205** | **REJECTED**  **N=284** | **P value** |
| --- | --- | --- | --- |
| Tracheotomized | 175 (85.4%) | 127 (44.7%) | **0.0001** |
| Extubated without tracheotomy | 13 (6.3%) | 144 (50.7%) | **0.0001** |
| Died without tracheotomy | 17 (8.3%) | 13 (4.6%) | 0.0912 |
|  |  |  |  |
| Weaning protocol applied in intubated patients | 43 (21%; 15-27) | 223 (78.5%; 73-83) | **0.0001** |
| Failed extubation (reintubated) | 24 (11.7%; 7-17) | 41 (14.4%; 10-19) | 0.3804 |
| Days of intubation in tracheotomized patients | 7 (5, 22) | 14 (9, 47) | **0.0000** |
| Days of intubation in successfully extubated patients | 14 (11, 17) | 10 (8, 21) | **0.0006** |
| Maximum PEEP | 7 (2, 16) | 7 (0, 16) | 0.1735 |
| Days free of MV (28 day) | 7 (0, 22) | 13 (0, 20) | **0.0005** |
| Days free of MV (90 days) | 69 (0, 84) | 75 (0, 82) | **0.0001** |
| Patients sedated nº (%) | 197 (96%; 92-98) | 272 (96%; 93-98) | 0.8588 |
| Deep sedation with neuro-blockers drugs | 46 (22%; 17-29) | 42 (15%; 11-19) | **0.0298** |
| Duration of sedation | 15 (2, 66) | 10.5 (0, 92) | **0.001** |
| Ventilation-associated Pneumonia | 23 (11%; 7-17) | 33 (12%; 8-16) | 0.8909 |
| Early complications of tracheotomy | 7 (1.4%; 1-7) | 4 (0.8%; 1-7) | 0.1399 |
| *Main reason for ventilatory support* |  |  |  |
| Acute respiratory insufficiency | 140 (68.3%; 61-74) | 203 (71.5%; 66-77) | 0.6375 |
| Neuro-muscular illness | 5 (2.4%; 1-6) | 4 (1%; 0.4-4) |  |
| Coma (CGS<10) | 47 (22.9%; 18-29) | 59 (21%; 16-26) |  |
| Decompensated COPD | 12 (5.9%; 3-10) | 13 (4.5%) |  |
| Acute asthma attack | 0 | 2 (1%; 0.1-3) |  |
| Other respiratory disease | 1 (0.5%; 0.1-3) | 3 (1%; 0.2-3) |  |

**Figure S1.** **Days of intubation until perform tracheotomy in randomized patients but previously rejected by attending physician (group 4).** TLI=trans laryngeal intubation
